# Engaging Stakeholders From Volunteer-Led Out-of-School Time Programs in the Dissemination of Guiding Principles for Healthy Snacking and Physical Activity

**DOI:** 10.5888/pcd12.150270

**Published:** 2015-12-24

**Authors:** Sara C. Folta, Alyssa Koomas, Nesly Metayer, Karen J. Fullerton, Kristie L. Hubbard, Stephanie Anzman-Frasca, Teresa Hofer, Miriam Nelson, Molly Newman, Jennifer Sacheck, Christina Economos

**Affiliations:** Author Affiliations: Alyssa Koomas, Nesly Metayer, Karen J. Fullerton, Stephanie Anzman-Frasca, Miriam Nelson, Jennifer Sacheck, Christina Economos, Tufts University, Boston, Massachusetts; Kristie L. Hubbard, Food and Nutrition Service, US Department of Agriculture, Western Regional Office, San Francisco, California; Teresa Hofer, City of Hope National Medical Center, Monrovia, California; Molly Newman, private practice, Boston, Massachusetts.

## Abstract

**Background:**

Little effort has focused on the role of volunteer-led out-of-school time (OST) programs (ie, enrichment and sports programs) as key environments for the promotion of healthy eating and physical activity habits among school-aged children. The Healthy Kids Out of School (HKOS) initiative developed evidence-based, practical guiding principles for healthy snacks, beverages, and physical activity. The goal of this case study was to describe the methods used to engage regional partners to understand how successful implementation and dissemination of these principles could be accomplished.

**Community Context:**

HKOS partnered with volunteer-led programs from 5 OST organizations in Maine, Massachusetts, and New Hampshire to create a regional “learning laboratory.”

**Methods:**

We engaged partners in phases. In the first phase, we conducted focus groups with local volunteer program leaders; during the second phase, we held roundtable meetings with regional and state program administrators; and in the final phase, we conducted additional outreach to refine and finalize implementation strategies.

**Outcomes:**

Implementation strategies were developed based on themes and information that emerged. For enrichment programs, strategies included new patch and pin programs that were consistent with the organizations’ infrastructure and usual practices. For sports programs, the main strategy was integration with online trainings for coaches.

**Interpretation:**

Through the engagement process, we learned that dissemination of the guiding principles in these large and complex OST organizations was best accomplished by using implementation strategies that were customized, integrated, and aligned with goals and usual practices. The lessons learned can benefit future efforts to prevent obesity in complex environments.

## Background

Out-of-school time (OST) presents an opportunity to foster healthy eating and physical activity habits among school-aged children. Structured, staff-led, after-school programs have been the target of several obesity prevention studies (1–3). However, little effort to date has focused on youth sports, scouting, and other volunteer-led OST programs, in which tens of millions of children participate each year (4). These programs are positioned to institute consistent opportunities for improved food choices and increased energy expenditure that may reduce the gap between ideal calories required for normal growth and excess calories that contribute to obesity (5). Changes in these settings can complement and bolster policies and programs in schools (6) and after-school programs (7). To help achieve such changes in volunteer-led OST programs, the Healthy Kids Out of School (HKOS) initiative was developed as part of ChildObesity180 at Tufts University, a portfolio of national initiatives that blends science and business strengths to prevent childhood obesity (ChildObesity180.org). HKOS proceeded in 5 stages:

1. Development of actionable guiding principles for healthy eating and physical activity with input from 9 national OST organizations (8)
2. Development of implementation and dissemination strategies regionally
3. Development and validation of evaluation tools (9)
4. Evaluation of effectiveness regionally
5. Implementation and evaluation nationwide

The guiding principles developed in our first stage were designed to be evidence-based, practical, and accessible: Drink Right: choose water instead of sugar-sweetened beverages; Move More: boost movement and physical activity in all programs; and Snack Smart: fuel up on fruits and vegetables. The next stage of HKOS was to support our partner OST organizations in disseminating the guiding principles to their local program leaders. To do this, we engaged regional OST partners in an iterative process to develop strategies for implementation. Here we describe that process and the resulting strategies.

## Community Context

The objective of the HKOS initiative was to increase opportunities for healthy snacking and physical activity in volunteer-led OST programs through dissemination of the guiding principles. Volunteer-led OST programs have many characteristics that affect adoption, including variability in access to resources, organizational structure, and communications channels. As volunteers, program leaders have limited time and a high turnover rate; therefore, training and implementation efforts must be simple and ongoing. To develop successful strategies to disseminate the guiding principles nationwide, we created a “learning laboratory” and partnered with volunteer-led OST programs in Maine, Massachusetts, and New Hampshire. In these 3 states, the rates of childhood obesity mirror national rates (10). Partners included Boy Scouts, 4-H, Pop Warner Football and Cheer, state youth soccer associations, and the youth sports programs in the YMCA. The focus of this initiative was on children aged 5 through 12. With approximately 25,000 clubs, troops, and teams, these organizations reach an estimated 330,000 children in the 3 states, including children from urban, rural, and low-income communities where there is an increased risk for childhood obesity. On the basis of publicly available data (11) and data provided to us by our partner organizations, we established that children in these programs reflect the general population in these states.

We engaged stakeholders to acquire diverse perspectives at the local, state, and regional levels. The decision to engage stakeholders was based on theoretical and practical considerations. From a theoretical perspective, stakeholder engagement is grounded in the key ethical concepts of participation, empowerment, fairness, and justice (12). At a practical level, stakeholder engagement can enhance uptake, because stakeholders are more likely to support initiatives that they have influenced (13); stakeholder engagement can also improve implementation, because it accounts for the realities and needs of those involved (14).

Engagement occurred in 3 phases (Figure). The overall objective of the process was to extend our knowledge of our partner organizations, develop implementation strategies, and refine plans for expanding reach. In the first phase, we conducted focus groups with volunteer program leaders such as 4-H club leaders and youth football coaches, with the objective of understanding barriers to and facilitators of using the guiding principles in local troop, club, and team meetings.

**Figure:**
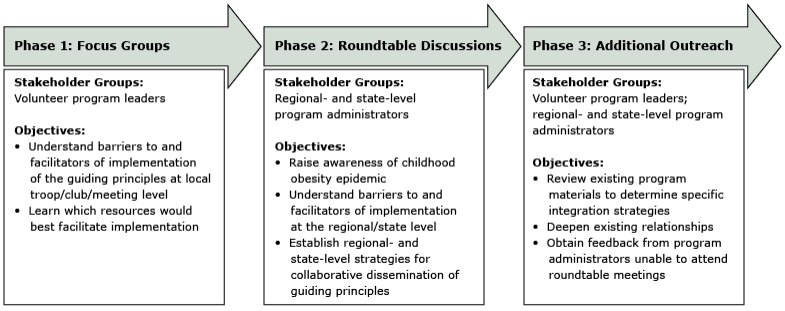
Three-phase process for engaging volunteer-led out-of-school-time organizations for the dissemination and implementation of guiding principles for obesity prevention, Maine, Massachusetts, and New Hampshire, 2012–2013.

In the second phase, we extended focus group findings through roundtable meetings with regional and state program administrators (eg, Pop Warner league presidents, Boy Scout executives, 4-H professionals). Depending on the organization, some regional or state administrators are paid staff. Program administrators are responsible for a range of functions, depending on the organization, including promoting the programs, registering local groups, training program leaders, and managing funds. The objective during this phase was to confirm focus group findings, develop tangible strategies, and begin dissemination of the guiding principles in partner programs. In the final phase, we conducted additional outreach to volunteer program leaders and administrators to further refine findings and finalize materials. This outreach included presentations, attendance at their organizations’ events, and key informant interviews. Research protocols were approved by the Tufts University Social, Behavioral and Educational Research Institutional Review Board.

## Methods and Outcomes

### Phase I: focus groups (November 2012–March 2013)

We conducted focus groups with program leaders to gain insights to help inform next steps in dissemination, which included gathering resources to populate the Healthy Kids Hub, a website designed to connect staff and volunteers with educational materials, tools, and other resources. Therefore, the primary objectives of the focus groups were to understand the facilitators of and barriers to implementing the guiding principles and resources we could provide to address them.

In total, we conducted 6 focus groups with program leaders from Boy Scouts, YMCA, 4-H, and Pop Warner Football and Cheer. Because of scheduling constraints, we could conduct focus groups only during youth soccer’s off-season, making recruitment of soccer coaches challenging. Each focus group comprised program leaders from a single organization, had 8 to 12 participants, and was less than 90 minutes long. In total, 56 program leaders participated. A pre–focus group survey was completed by participants to gather data on demographics, length of time associated with their organization, and preferred channels for communication. The mean age of participants was 42 years (range, 22–82 y). Sixty percent of participants were female; 80% were non-Hispanic white, and 66% had worked in the OST organization for 5 or more years. The data analysis followed the accepted practices of grounded theory approach (15). Three researchers (K.L.H., N.M., and T.H.) conducted multiple readings of the focus group transcripts and organized emerging themes into a data display for each group. Constant comparison was used to reconcile findings until consensus was reached on a final display. Data were reviewed and the findings confirmed by 5 members of the research team (S.C.F., A.K., K.L.H., N.M, and K.J.F.) responsible for the focus groups.

Focus group participants indicated that implementation of the Drink Right and Move More guiding principles would be easier and more relevant than Snack Smart. Move More was already part of the mission of some groups, and many viewed Drink Right as already being implemented (“We always drink water. Push water all the time when we’re out hiking, you know? That one is easy.” [Boy Scout leader]). Barriers to Snack Smart were cited at the organizational level and included policies, existing practices, reliance on partnerships to finance healthy snacks, food insecurity or poverty, and the influence of food advertising. Children’s preferences for less healthy foods and lack of interest in physical activity were also mentioned as barriers to implementation of the guiding principles.

We identified themes related to factors that would facilitate implementation of the guiding principles. These included program assets such as supportive organizational policies, formal infrastructure for training and leadership, and strategic partnerships. Program leaders also expressed an interest in learning more about children’s health and indicated that the guiding principles would largely integrate into their existing values, which included promoting the health and well-being of the children they served.

During the focus groups, participants were prompted to describe the types of materials and resources that would be the most helpful for implementing the guiding principles. They expressed interest in 3 types of resources: 1) discounted products (eg, grocery store vouchers for fruits and vegetables, sports equipment), 2) educational materials targeting parents (eg, recipes, games), and 3) mechanisms for connecting with other program leaders (eg, sharing resources and ideas) and community resources to support the guiding principles (eg, dietitians, Zumba instructors).

Feedback from the focus groups indicated that a dissemination strategy that put the burden on individual leaders to champion adoption of the guiding principles in the organization could be problematic, especially in programs that depended heavily on unpaid volunteers for administration and operations (ie, 4-H and Pop Warner Football and Cheer). This feedback influenced us to develop a plan that would use existing communication and training channels and that would institute policy changes in the organizations, such as a youth soccer requirement that all new coaches take a HKOS-developed online healthy habits training.

### Phase II: roundtable discussions (March 2013)

In Phase II, we engaged program administrators working at the state or regional level in half-day roundtable discussions with the objectives of raising awareness about the childhood obesity epidemic and the purpose of the guiding principles. We further aimed to establish state- and regional-level strategies for collaborative HKOS implementation and dissemination.

One roundtable meeting was held at a central location in each state. At the Maine roundtable, 10 of 16 invited program administrators attended; in New Hampshire, 7 of 12 invited administrators attended; and in Massachusetts, 12 of 30 invited administrators attended. In each state, a national YMCA representative also attended. Youth soccer was not represented in New Hampshire, because there was only one state program administrator at the time, who was unable to attend; Pop Warner was not represented in Maine, because this program is not active in that state. For administrators who expressed financial barriers to attendance, we offered a stipend of $150 to offset travel costs.

During the roundtable meetings, we provided program administrators with background information on the childhood obesity epidemic as well as evidence supporting OST programs’ role in obesity prevention. We held a facilitated discussion to garner their reactions to the information presented and to learn their perceptions of the barriers to and facilitators of implementing the guiding principles. Feedback clustered around 3 major themes. First, dissemination would likely be facilitated by the simple, actionable nature of the guiding principles. Second, there was concern about the potential loss of revenue from extending the Drink Right and Snack Smart principles to food environments outside of regular program meetings, such as concessions stands, Boy Scouts “trading posts,” or camp stores. Third, participants said that it would be most effective to reach program leaders through their existing training and communications channels, confirming a major theme from the focus groups. Program administrators also indicated that the information we provided was a powerful motivator and should also be shared with program leaders, because it challenged common assumptions. For example, we had shared research findings that children are physically active for only about half the time during a typical sports practice (16,17). The administrators supported the idea of integration of materials into existing in-person and online trainings to maximize reach and minimize the burden on program leaders. In addition to these themes, roundtable meetings helped cultivate our vital relationships with state and regional leaders that facilitated the ensuing collaboration.

### Phase III: additional outreach (April–June 2013)

The focus groups and roundtable discussions provided a more in-depth understanding of each organization that guided our dissemination strategy of tailoring the guiding principles to each organization’s unique programming and communication channels. Our main objective in this phase was to obtain information and feedback from program leaders and review existing program materials to determine strategies for integrating messages into them. We also aimed to deepen existing relationships and engage with any program administrators who were unable to attend the roundtable discussions.

Our team delivered presentations during our OST partners’ regional meetings, attended OST events, and conducted key informant interviews (by telephone) with program leaders and administrators. For example, regionally we made presentations at several events, including a New England Region Pop Warner Football and Cheer League President’s meeting and a New England Area Scout Executive’s meeting. At the local level, HKOS hosted booths and delivered presentations at events, such as a Boy Scout Council’s Chuckwagon Derby and a youth soccer league tournament. In these settings, we engaged troop leaders, coaches, and youth in interactive activities related to the guiding principles, promoted adoption of them, and gathered additional feedback on the best way to reach program leaders with messages and materials. Once connections were made, these program leaders became advisors on future materials and plans. For example, an additional focus group was held with 4-H program leaders to obtain feedback on content and usability of the Healthy Kids Hub website.

During the final phase of engagement, major differences among and within the organizations were identified, including the variability of program leader training (eg, one-time vs continuous, optional vs mandatory) and communication channels (ie, in-person meetings, online training courses, printed materials, emails, and newsletters). This information reinforced the need to customize our approach and to integrate strategies into existing structures. As a result, organizational variability was recognized and respected, extra demands on program leaders’ time were limited, and dissemination of the principles was relevant to the organizational context.

### Implementation strategies

For enrichment programs, the main implementation strategies resulting from the engagement process were trainings that promote activities consistent with the organization’s infrastructure or usual practices, such as youth earning a patch or pin on meeting goals related to the guiding principles (Table). For the sports programs, the main strategy was development of a video training on the principles to integrate with existing online trainings for coaches. To support these strategies, we aimed to make custom materials that were relatable, actionable, and measurable. For example, we worked with the Boy Scouts of America national office to incorporate the snack and physical activity principles into weekly meeting times by creating the SCOUTStrong Healthy Unit patch that aligns with the SCOUTStrong Healthy Living Initiative. We modeled the tracking of steps to earn the new patch on existing scouting materials. We provided background to highlight the importance of the behavioral change, related it to an existing national scouting program, and then demonstrated steps that volunteer program leaders could take. To promote this new patch to volunteer program leaders, we developed a training packet that was presented in person during monthly district roundtable meetings.

**Table:** Implementation Strategies for the Healthy Kids Out of School Guiding Principles in Maine, Massachusetts, and New Hampshire, 2012–2013

OST Organization	Primary Implementation Strategy
Boy Scouts	SCOUTStrong Healthy Unit Patch materials disseminated via in-person district roundtable meeting presentations (approximately 30 min). Actions promoted: • Fruit or vegetable served at 3 meetings • Water served at 6 meetings • 15 min of physical activity at 9 meetings
4-H	4th H for Health Challenge materials disseminated via in-person 4-H Leader and Advisory Council meetings at the county level (approximately 45 min). Actions promoted: • Fruit or vegetable served at 4 meetings • Water served at 4 meetings • 15 min of physical activity at 4 meetings • Choose one or more of the above at 4 additional meetings
Youth soccer	Coaching Healthy Habits online coaches’ training module (12 min) disseminated via Massachusetts Youth Soccer website and the Healthy Kids Hub website. Completion of training is required for all coaches receiving a license in Massachusetts, New Hampshire, and Maine and voluntary for all others. Actions promoted: • If snacks are provided, make them fruits or vegetables • Conduct “water only” practices • Increase active time during practices by 10 to 15 min
Pop Warner Football and Cheer	Coaching Healthy Habits online coaches’ training module (8 min) disseminated via the Healthy Kids Hub website and promoted during in-person presentations at league meetings, Facebook posts, and email blasts. Completion of training is voluntary. Actions promoted same as for youth soccer.
YMCA youth sports	Coaching Healthy Habits online coaches’ training module (8 min) disseminated via the Healthy Kids Hub website and promoted via YMCA executive director and sports directors. Whether completion of training is required or voluntary varies by YMCA association. Actions promoted same as for youth soccer.

Abbreviation: OST, out-of-school time.

## Interpretation

Information learned from the engagement process was used to develop strategies for implementation of the HKOS guiding principles that can improve the likelihood of their successful dissemination. We learned that it is important to customize strategies and materials to align with the organizations’ usual practices, goals, and values.

Integration of the custom materials into leader trainings and organizational policies increased the potential for sustainability. Incorporating the guiding principles into new leader trainings (eg, online coach’s training module) and handbooks (eg, Boy Scouts leader handbook) may cause a shift in cultural norms that could affect the next cohort of program leaders and gain momentum in the future. Ideally, this approach removes the onus from program leaders to adopt the guiding principles and makes the behaviors synonymous with participation in the program.

The results of our engagement process further suggested that dissemination would be most effective by reaching program leaders through their organizations’ existing communication channels. This process worked best when the HKOS team proposed and developed customized resources and then sought feedback at the program level for refinement. The SCOUTStrong Healthy Unit patch is a clear example of a tailored resource that was integrated into the existing SCOUTStrong program in the OST organization and disseminated through the existing communication channel of district roundtable meetings.

Development of the customized materials and online training modules required the investment of substantial time and resources. The HKOS team that worked on this regional effort comprised a full-time project manager, 2 full-time research coordinators, and several Tufts University staff and faculty members who provided guidance and input on all aspects of the initiative. The potential reach justifies the more intensive inputs; our success with regional programs has led to interest in and adoption of our materials and trainings at the national level of our partner organizations and in similar OST organizations (eg, Girl Scouts). The materials and new leader training modules developed in this regional project have been promoted on the national websites of 4 of the 5 partnering organizations, with the potential to affect millions of children. Therefore, our iterative process of engagement was successful in allowing us to navigate and affect multiple levels of these large and complex national organizations. By necessity, the development and initial adoption of the guiding principles occurred at the national level, effectively a “top-down” approach. Through the engagement process in the 3 states, we were able to integrate this approach with “bottom-up” strategies for successful local implementation and dissemination.

Although this work was conducted in volunteer-led OST organizations, we believe the approach to engagement and the lessons learned could be applied to many endeavors to disseminate consistent evidence-based principles across multiple organizations. Important insights were gained with each iteration and by engaging more than one organizational level. This engagement process may generalize to many other types of organizations that are similarly complex. Our findings about the importance of customization and integration are also likely to generalize to other settings, including nonvolunteer organizations, given the ubiquitous challenges related to staff turnover and limited resources, as well as the importance of stakeholder ownership and support in enhancing adoption and implementation (14).

The guiding principles are a powerful, policy-oriented approach to obesity prevention. They are a mechanism for unifying multiple OST organizations around consistent public health goals in a way that can complement efforts in other environments that children encounter. The stakeholder engagement process provided multiple insights to enhance dissemination of the guiding principles in this setting.
